# Status Epilepticus in Immature Rats Is Associated with Oxidative Stress and Mitochondrial Dysfunction

**DOI:** 10.3389/fncel.2016.00136

**Published:** 2016-05-26

**Authors:** Jaroslava Folbergrová, Pavel Ješina, Hana Kubová, Rastislav Druga, Jakub Otáhal

**Affiliations:** Institute of Physiology, The Czech Academy of SciencesPrague, Czech Republic

**Keywords:** immature rats, status epilepticus, oxidative stress, mitochondrial dysfunction, brain damage, protection

## Abstract

Epilepsy is a neurologic disorder, particularly frequent in infants and children where it can lead to serious consequences later in life. Oxidative stress and mitochondrial dysfunction are implicated in the pathogenesis of many neurological disorders including epilepsy in adults. However, their role in immature epileptic brain is unclear since there have been two contrary opinions: oxidative stress is age-dependent and does not occur in immature brain during status epilepticus (SE) and, on the other hand, evidence of oxidative stress in immature brain during a specific model of SE. To solve this dilemma, we have decided to investigate oxidative stress following SE induced in immature 12-day-old rats by three substances with a different mechanism of action, namely 4-aminopyridine, LiCl-pilocarpine or kainic acid. Fluoro-Jade-B staining revealed mild brain damage especially in hippocampus and thalamus in each of the tested models. Decrease of glucose and glycogen with parallel rises of lactate clearly indicate high rate of glycolysis, which was apparently not sufficient in 4-AP and Li-Pilo status, as evident from the decreases of PCr levels. Hydroethidium method revealed significantly higher levels of superoxide anion (by ∼60%) in the hippocampus, cerebral cortex and thalamus of immature rats during status. SE lead to mitochondrial dysfunction with a specific pronounced decrease of complex I activity that persisted for a long period of survival. Complexes II and IV activities remained in the control range. Antioxidant treatment with SOD mimetic MnTMPYP or peroxynitrite scavenger FeTPPS significantly attenuated oxidative stress and inhibition of complex I activity. These findings bring evidence that oxidative stress and mitochondrial dysfunction are age and model independent, and may thus be considered a general phenomenon. They can have a clinical relevance for a novel approach to the treatment of epilepsy, allowing to target the mechanisms which play a crucial or additive role in the pathogenesis of epilepsies in infants and children.

## Introduction

Epilepsy is common neurologic disorder which is particularly frequent in infants and children where it can lead to serious consequences, concerning brain maturation, brain damage, and neurocognitive dysfunctions (Sankar et al., 1998; Jensen, 1999; Lynch et al., 2000; Kubová et al., 2001, 2004; Wasterlain et al., 2002). Current therapies are mainly symptomatic and thus ineffective. The novel therapeutic targets and strategies must therefore be revealed.

Increasing evidence shows that oxidative stress and mitochondrial dysfunction are implicated in the pathogenesis of many neurological disorders, including epilepsy in adults (Patel, 2004; Lin and Beal, 2006; Waldbaum and Patel, 2010; Folbergrová and Kunz, 2012; Rowley and Patel, 2013). However, their role in immature epileptic brain is unclear. So far, the consensus opinion (based on KA model) has been that oxidative stress during SE is age-dependent and does not occur in immature brain (Bruce and Baudry, 1995; Patel and Li, 2003; Sullivan et al., 2003). We have recently shown (using HCA – induced seizures), that oxidative stress does occur during SE in immature brain (Folbergrová et al., 2007, 2010, 2012). In addition, neuronal degeneration demonstrated in this model (Folbergrová et al., 2005) was significantly attenuated following treatment with the spin trapping agent *N*-*tert*-butyl-α-phenylnitrone (PBN; Folbergrová et al., 2006). Finally, the marked decrease of mitochondrial complex I activity could be substantially prevented by treatment with selected free radical scavengers, namely SOD mimetics Mn (III) tetrakis (1-methyl-4-pyridyl) porphyrin pentachloride (MnTMPYP) and 4-hydroxy-2,2,6,6-tetramethylpiperidine-1-oxyl (Tempol) or a selective peroxynitrite scavenger and decomposition catalyst FeTPPS (Folbergrová et al., 2007, 2010). Altogether, these data arise fundamental question on, whether oxidative stress and mitochondrial dysfunction observed in brain of immature rats following HCA-induced seizures is a peculiarity of this specific model. In order to address this issue, we have designed a more extensive study such as to verify the possibility that oxidative stress in immature brain during seizures is universally present.

The aim of the present study was to evaluate oxidative stress and mitochondrial dysfunction after SE induced in immature 12-day-old rats by three compounds bearing a different mechanism of action. Specifically, we used the potassium channel blocker 4-AP, the cholinergic muscarinic agonist pilocarpine (Li-Pilo) and KA. In this study we particularly aimed:

(1) To characterize the employed models of generalized seizures, regarding behavior pattern, EEG recordings and the energy status.
(2) To determine $O_{2}^{•–}$ formation in different brain regions, following 60 min lasting SE.
(3) To measure the activity of mitochondrial complexes, particularly of complex I.
(4) To compare the character of brain damage 24 h after acute phase of seizures.
(5) To evaluate the potential protective effect of selected free radical scavengers.

Our data revealed that oxidative stress does occur in the immature brain during SE and it thus plays an important role in the pathophysiology of epilepsy during early stages of brain development. Importantly, it can have a clinical relevance for a novel approach to the treatment of epilepsy, suggesting potential benefit from the use of substances with antioxidant properties combined with conventional therapies.

## Materials and Methods

### Animals

Immature 12-day-old male Wistar rats were used for these experiments. Twelve-day-old rats were chosen because of the level of brain maturation which is comparable to the early postnatal period in human infants (Dobbing, 1970). The rat pups were removed from their dams 1 h before the experiment. They were kept in plastic observation chambers on an electrically heated pad at 34°C (i.e., the temperature of the nest), with the exception of surgery. The protocol of experiments was approved by the Animal Care and Use Committee of the Institute of Physiology, Academy of Sciences of the Czech Republic, in agreement with Animal Protection Law of the Czech Republic, which is fully compatible with the guidelines of the European Community Council directives 86/609/EEC. The Institute possesses The Statement of Compliance with Standards of Humane Care and Use of Laboratory Animals #A5228-01 from NIH. All efforts were made to minimize animal suffering and to reduce the number of animals used.

### Surgery

**FIGURE 1:**
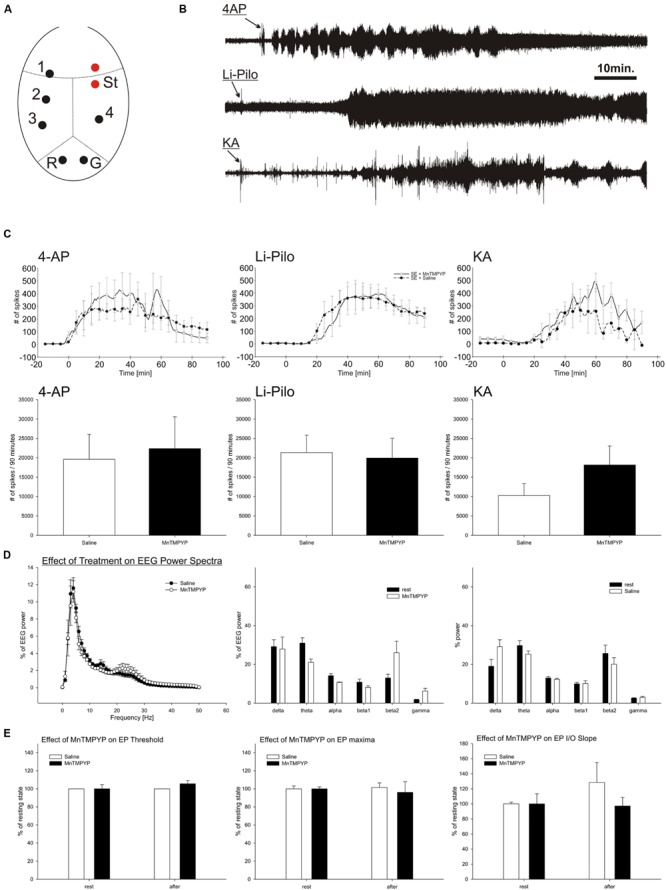
**EEG activity during SE induced by 4-AP, Li-Pilo and KA **(B,D)**.** Influence of MnTMPYP on number of spikes during SE **(C)** and evoked potentials **(E)**. Electrode placement is depicted on panel **(A)**. For more details see section “Materials and Methods.”

The animals were anaesthetized with isoflurane and fixed in a stereotaxic apparatus, modified for rat pups (Folbergrová et al., 2000). For 4-AP application, bilateral stainless steel guide cannulae (26-gage, 4 mm in length, Plastics One, Germany) were stereotaxically implanted 1 mm above the lateral ventricles (AP:0.7 mm caudal from the bregma; L: ±1.5 mm; V: 3.3 mm from the skull surface). For EEG recordings silver electrodes were inserted through series of tiny craniotomies into epidural space. To record spontaneous EEG, a pair of recording electrodes was placed bilaterally over the sensorimotor cortex. For evoked potentials, three recording electrodes were placed on the left and one on the right side above the sensorimotor cortex. Two stimulation electrodes were placed above the right motor cortex 2 mm lateral and -1 and 1 mm rostro-caudally to bregma (**Figure 1A**). Reference and ground electrode were placed into the occipital bone above the cerebellum. Electrodes and/or cannulae were fixed to the skull with dental acrylic. After the surgery animals were returned to their mothers in home cages to recover.

### Seizure Induction and Treatment

Four-AP (Sigma–Aldrich) was dissolved in sterile saline and the pH adjusted to ∼7.0, only freshly prepared solutions were used. Bilateral i.c.v. infusions of 4-AP (100 nmol/side; or saline) were made in a volume of 0.5 μl at a rate of 0.17 μl/min using a SP200i infusion pump (WPI, USA) through stainless steel internal cannulae (33-gage, 5 mm in length, Plastics One, Germany), each connected by a polyethylene tube to a 10 μl Hamilton syringe. To induce Li-Pilo SE, LiCl (Sigma–Aldrich) was dissolved in redistilled water and administered i.p. to PD11 immature rats (127 mg/kg). After 24 h, pilocarpine (Sigma–Aldrich), dissolved in redistilled water was given i.p. (35 mg/kg) to lithium pretreated pups (Hirsch et al., 1992). To induce kainate SE, KA (Tocris Bioscience, Bristol, UK) was dissolved in saline and given i.p. (6 mg/kg). Control animals received corresponding volumes of the appropriate vehicles.

For evaluating a potential protective effect, SOD mimetic MnTMPYP (from Calbiochem) and a selective peroxynitrite scavenger and decomposition catalyst FeTPPS (from Calbiochem) were employed. Scavengers were dissolved in saline (MnTMPYP) or in redistilled H_2_O (FeTPPS) and the resultant solutions were neutralized to pH ∼7.0 when necessary. Both scavengers were given by i.p. injections in two doses; MnTMPYP (3 mg/kg each), one 30 min prior and the second 30 min after application of convulsant drugs and FeTPPS (10 mg/kg each), one 90–120 min prior and the second 6–10 min after convulsant drugs application. Doses and time schedule for application were based on our previous studies (Folbergrová et al., 2007, 2010).

### EEG Recordings and Analysis

After more than 1 h postsurgery recovery, animals (comprising always four pups with convulsant agent alone and four pups with convulsant agent plus MnTMPYP) were connected to Video-EEG monitoring system (Pentusa, TDT, USA) and spontaneous EEG was continuously recorded for 120 min. During the whole experiment the temperature of an animal cage was kept constant using heating pad. Number and frequency of spikes and power spectra were analyzed off-line using custom written scripts in Matlab software (Mathworks, USA). EEG spectral bands were defined to fit to rat EEG (delta 0 to 4 Hz, theta 4 to 8 Hz, alpha 8 to 12 Hz, beta1 12 to 16 Hz, beta2 16 to 32 Hz, gamma 32 to 50 Hz).

To evaluate cortical excitability, the part of the animals (*n* = 8) underwent evoked potential testing. The cortical evoked potentials were elicited by biphasic constant current stimulation of the contralateral sensorimotor cortex before and after MnTMPYP (*n* = 4) or saline (*n* = 4) application. Amplitude of the transcallosal monosynaptic response was measured and Input-Output curve constructed.

### Energy Metabolites

The animals were randomly selected into two experimental groups (five animals in each group) comprising animals with 4-AP, Li-Pilo and KA seizures, respectively, and the corresponding controls. The rat pups were rapidly frozen in liquid nitrogen during the period of generalized seizures, approximately 45–60 min after application of convulsant compounds. The brains were dissected out in a glove box at -22°C. Samples of the cerebral cortex (weighing approximately 25–28 mg) were extracted at -30°C with HCl/methanol and subsequently at 0°C with perchloric acid, as described in more detail previously (Folbergrová et al., 1972). Energy metabolites, PCr, ATP, glucose, glycogen and lactate, were determined by enzymatic fluorimetric methods according to Lowry and Passonneau (1972).

### Superoxide Anion Determination

Formation of $O_{2}^{•–}$ in different brain regions *in situ* was determined using hydroethidium (Het) method (Bindokas et al., 1996) adopted for immature rats (Folbergrová et al., 2012). Stock solution of Het (from Molecular Probes; 100 mg/ml in DMSO) was diluted to 1 mg/ml by DMSO + PBS (final concentration of DMSO ∼22%). Het was given by i.p. injection immediately before infusion of 4-AP and ∼15 min after i.p. administration of Li-Pilo or KA (final concentration 10 mg/kg). One hour after the application of Het, rat pups were deeply anesthetized with 20% (w/v) urethane and transcardially perfused with 0.01 M phosphate buffered saline (PBS), pH 7.4, followed by a fixative solution [4% (w/v)] paraformaldehyde in 0.1 M phosphate buffer, pH 7.4. The brains were removed from the skull, postfixed for 3 h at 4°C in the same fixative, then cryoprotected in sucrose of increasing concentrations [10, 20, and 30 % (w/v), respectively] in 0.1 M phosphate buffer, pH 7.4 and finally frozen in dry ice. Coronal sections (50 μm) were cut through the brain in a cryostat and mounted onto the gelatinated slides. All procedures were performed in the dark.

The determination of the oxidized products of Het was assessed microscopically by detection of their fluorescence (>600 nm). Pictures of the selected regions of interest (hippocampal fields CA1, CA3, and DG, primary somatosensory cortex and dorsal thalamus) of the same size and orientation, were captured (AP -3.5 to -4.0 according to Paxinos and Watson, 1998) with cooled camera mounted onto upright microscope (10× magnification lens). Camera settings remained unchanged throughout the evaluation of the current set of tissue sections of animals from one experimental day, treated with the same solution of Het. This set of animals comprised always at least three saline-treated controls, three animals with convulsant drug alone and three with convulsant drug plus the tested scavenger. Fluorescence signal (represented as integral intensity of the given region) was normalized by values of the control animals of the current set. Results are expressed as percentage of saline-treated animals.

### Isolation of Mitochondria

Mitochondrial fractions were isolated using the method of Liang et al. (2000). All procedures for mitochondrial isolation were conducted at 4°C. Cerebral cortices (weighing ∼250 mg) were used for each mitochondrial preparation. 10% (w/v) homogenates in ice-cold mitochondrial isolation buffer (70 mM sucrose, 210 mM mannitol, 5 mM Tris-HCl, 1 mM EDTA, pH 7.4) were prepared with Elvehjem-Potter type glass-Teflon homogenizers manually by twenty slow up-and-down strokes. Homogenates were centrifuged at 600 × *g* for 5 min at 4°C, the postnuclear supernatant was centrifuged at 17 000 × *g* for 10 min at 4°C. Mitochondrial pellet was resuspended with 100 μl 50 mM Tris-HCl (pH 7.4). Fresh isolated mitochondria were used for protein determination. Aliquots of mitochondria frozen in liquid nitrogen and stored at -70°C were used for complex activities measurements that were performed within 1 week.

### Enzyme Assays

Activities of mitochondrial respiratory chain complexes and citrate synthase were measured at 30°C in a total reaction volume of 1 ml using Shimadzu 1601 spectrophotometer. Duplicate determinations were carried out with each mitochondrial sample. More details of the assay conditions for complexes I, II, and IV and for citrate synthase are described in our previous studies (Folbergrová et al., 2007, 2010).

Activity of individual complexes was expressed as nmol/min/mg protein. To correct for the potential variations in mitochondrial contents in the samples, mitochondrial chain complex activities were also expressed as a ratio to citrate synthase.

### Protein Determination

Mitochondrial protein concentration was estimated by Bradford’s method, using bovine serum albumin as a standard.

### Brain Damage

For the evaluation of brain damage, at 24 h following the SE rat pups were subjected to the same fixation procedure as that described above. Coronal sections (50 μm) were used for Nissl and Fluoro-Jade B staining as described in our previous papers (Folbergrová et al., 2005, 2006, 2008). Fluoro-Jade B stained sections were examined using an Olympus AX 70 fluorescent microscope (filter cube for fluorescein, excitation band 450–490 nm, and emission band above 515 nm). Photomicrographs were taken with a digital camera Olympus DP-70. After gross neuropathological inspection neurodegeneration was assessed in the selected thalamic nuclei, in the septal as well as temporal part of the hippocampal formation (fields CA1, CA3, and DG), in the septum, hypothalamus, and in the complex of amygdalar nuclei and expressed in semiquantitative scale (0–4 neurons per area of evaluated structure = 0; 5–10 neurons = 1; 11–20 neurons = 2; 21–40 neurons = 3; more than 40 = 4).

### Statistics

The data were evaluated by one-way ANOVA with Newman–Keul’s *post hoc* test or by *t*-test where appropriate. The level of statistical significance was set to 5%.

## Results

### General Description of the Behavioral Pattern of Seizures

All three convulsants induced SE which was characterized in 4-AP model by generalized clonic-tonic seizures, in Li-Pilo model by generalized clonic seizures and in KA model by generalized clonic seizures, accompanied by mild tonic extensions.

### 4-AP

Shortly upon icv infusion of 4-AP, a typical behavioral pattern began with “dog shakes” of the head followed by the whole body. Intensity gradually increased and approximately after 10–15 min clonus of one or both forelimbs could be observed. Clonus was usually followed by a fall of the animals to either side, but this was immediately corrected by the pups themselves. The intensity of motor phenomena increased, with wild running followed by falling of the animals to either side with clonic movements of all extremities. This phase was usually followed by tonic extension of the forelimbs, most often also hindlimbs. Animals displayed repeated clonic-tonic convulsions for ∼90–120 min, after which the frequencies and intensity gradually diminished. After 24 h rat pups seemed to have recovered. The mortality rate was relatively low since ∼80% of animals survived.

### Li-Pilo

Following the injection of pilocarpine, there was a latent period of approximately 8–10 min, after this animals became restless, with occasional scratching and trembling, followed by the occurrence of clonic movements of one or later in both forelimbs, sometimes with rearing. Intensity and frequency of clonic seizures gradually increased, in many animals accompanied by falling on either side. Inspite of a pronounced intensity of clonic convulsions, no signs of tonic extension and/or flexion could be observed. Mortality rate was approximately 10%.

### KA

In this model, after approximately 8–10 min lasting latent period, rat pups began with scratching of hind limbs, followed by the first signs of clonic movements of forelimbs. The intensity and frequency of clonic movements gradually increased, followed by falling animals to either side, this was in the early phase corrected by the pups themselves. Later, approximately after 30–40 min animals remained to lie on side, with intense clonic movements of all extremities, and with typically splayed fingers on forelimbs. There was occasionally a sign of tonic extension, but this remained uncomplete. SE cotinued for about 2–3 h folowed by a gradual recovery that seemed after 24 h as complete. Mortality concerned approximately 20% of animals.

### Effect of MnTMPYP and FeTPPS on Behavioral Pattern of SE

In all three models, both latency and character of SE were not influenced by MnTMPYP or FeTPPS. Also mortality was not changed by these scavengers.

### Effect of MnTMPYP on Spontaneous EEG Activity and Cortical Excitability

Total power and power spectra of the baseline EEG corresponded to the electrode placement and age of the animals (**Figure 1D**). MnTMPYP tended to increase high frequency bands of the EEG (beta2 and gamma, **Figure 1D**) while the total EEG power was slightly lower than in saline treated animals (55 ± 5 and 78 ± 13% of baseline power, respectively).

Suprathreshold stimulation of the motor cortex elicited a typical evoked potential recorded on contralateral hemisphere. The analysis of the first monosynaptic response was performed to elucidate an effect of MnTMPYP on cortical excitability. The threshold and stimulation intensity for maximal response remained unaffected regardless the treatment (**Figure 1E**). Average latency of the response (33.6 ± 0.9 ms) was not altered either by saline (33.4 ± 1.8 ms) or MnTMPYP (33.7 ± 0.2 ms). The slope of the input–output curve which is a measure of excitability did not differ between the groups (**Figure 1E**).

### Effect of MnTMPYP on Electrographic Activity during Experimentally Induced SE

Intracerebroventricular application of 4-AP led immediately to occurrence of epileptic discharges and occurrence of recurrent clonic-tonic seizures which were interrupted by short periods of EEG suppression. Similarly, KA induced epileptic discharges in EEG with latency 21 to 25.5 min followed by recurrent clonic-tonic seizures. In contrast, pilocarpine elicited epileptic discharges after 28–30min after the application. The character of pilocarpine SE differed. When animals reached the grades 3–4 of Racine scale the clonic epileptic activity remained mostly uninterrupted which corresponds to continuous electrographic seizures (**Figures 1B,C**).

The treatment with MnTMPYP did not affect latency to occurrence of epileptic discharges nor spike frequency or count in a 90 min analysis period. Severity of behavioral or electrographic seizures was not significantly modified by MnTMPYP treatment. However, in KA model it had tendency to worsen the epileptic activity as revealed by non-significant increase in spike number (**Figures 1B,C**).

### Energy Metabolite Changes during Seizures

Selected energy metabolites were examined in the cerebral cortex of immature rats during the acute phase of seizures, lasting ∼45–60 min.

### Phosphocreatine (PCr)

**FIGURE 2:**
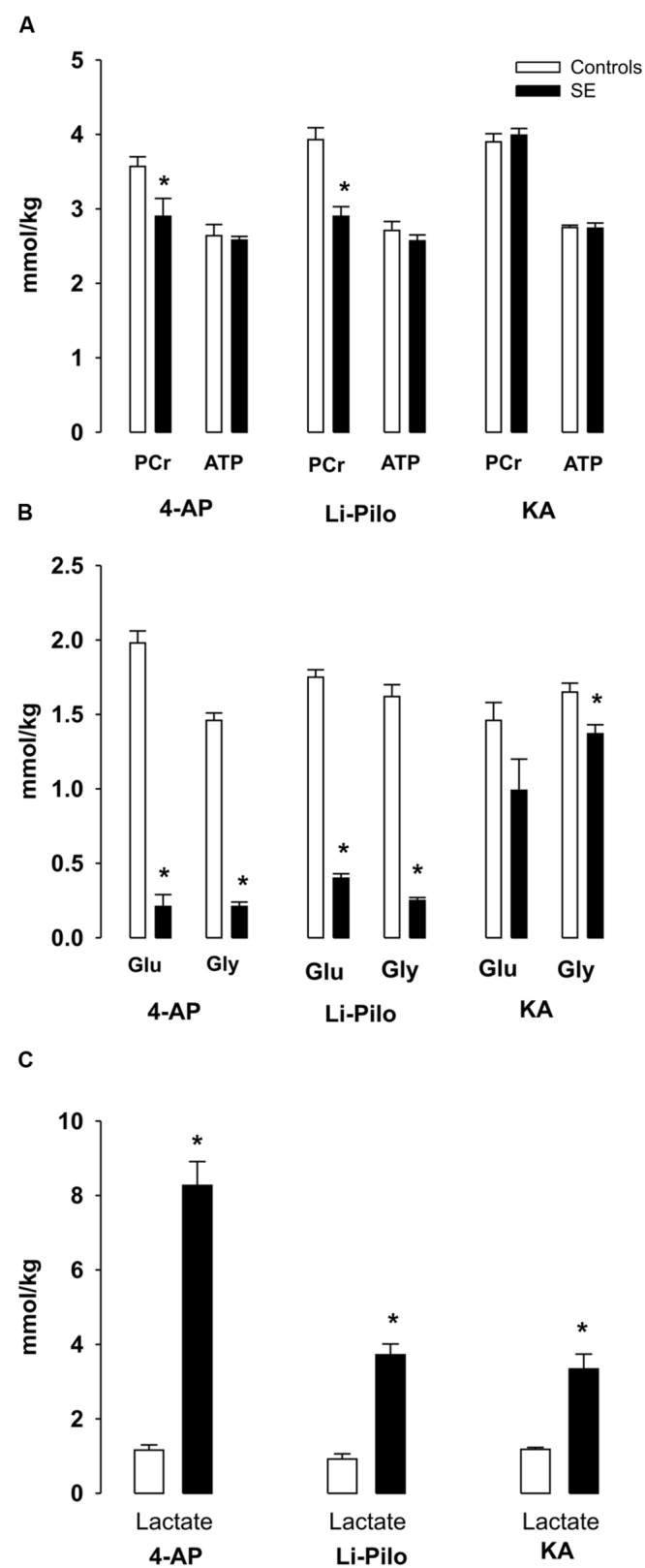
**(A–C)** Metabolic changes in cerebral cortex of immature rats during SE induced by 4- AP [bilateral i.c.v infusion, (100 nmol/side)], Li-Pilo [i.p. administration of LiCl (127 mg/kg)] followed the next day by pilocarpine (35 mg/kg) and KA [i.p. administration (6 mg/kg)]. Rat pups were sacrificed during the period of generalized seizures, ∼45–60 min after application of convulsant compounds. White columns, vehicle-treated controls; black columns, seizure groups; Glu, glucose; Gly, glycogen. Results are expressed in mmol/kg wet wt., mean values for 5 animals ± SEM. ^∗^*P* < 0.05 as compared with the control animals.

There was a small, but significant decrease of PCr levels during 4-AP -and Li-Pilo – induced SE (∼18.8 and 26%, respectively). In KA model PCr levels remained unchanged (**Figure 2A**).

### ATP

ATP levels remained unchanged in all three models studied (**Figure 2A**).

### Glucose, Glycogen, and Lactate

4-AP- and Li-Pilo-induced SE was accompanied by a large decreases of glucose and glycogen (**Figure 2B**), whereas in KA model glucose levels did not significantly change as compared with control values and there was only a small, but significant decrease (∼17%) of glycogen (**Figure 2B**). Lactate levels increased in all three models, but to a different extent, during 4-AP SE approximately sevenfold, in Li-Pilo model about 4- times and in KA model less than 3- times (**Figure 2C**).

### Generation of $O_{2}^{•–}$ during Seizures

**FIGURE 3:**
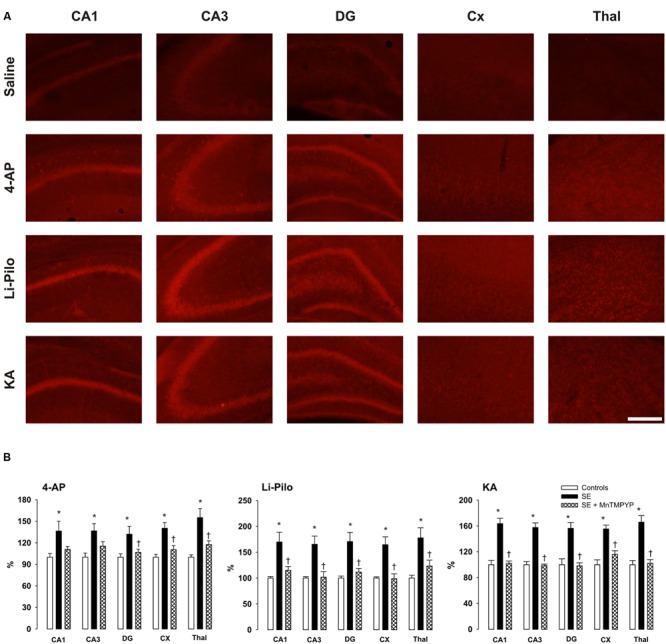
**(A)** Fluorescence of the oxidized products of hydroethidium (reflecting $O_{2}^{•–}$ production), assessed microscopically by fluorescence (>600 nm) in various brain structures following 60 min lasting SE induced in immature rats by 4-AP, Li-Pilo or KA (for more details see Material and Methods). **(B)** Effect of SOD mimetic MnTMPYP on $O_{2}^{•–}$ formation at 60 min following the onset of SE. MnTMPYP was given by i.p. injections in two doses (3 mg/kg each), one 30 min prior and the second 30 min after the application of convulsant compounds. White columns, saline-treated controls; black columns, convulsant agent alone; cross-hatched columns, convulsant agent plus MnTMPYP. Results are expressed in percent, compared to 100% in the control animals. Mean values for 5–6 animals ± SEM. ^∗^*P* < 0.05 as compared with saline; ^†^*P* < 0.05 as compared with convulsant compounds alone. Scale bar indicates 500 μm.

The fluorescent signal of the oxidized products of Het (reflecting $O_{2}^{•–}$ production) significantly increased in all the studied structures, namely CA1, CA3, and DG of hippocampus, cerebral cortex and thalamus of immature rats after 60 min lasting seizures induced by all three convulsants (**Figure 3A**). This increase corresponds to ∼32–55% in 4-AP model, ∼55–65% in KA-induced and ∼65–75% in Li-Pilo-induced SE (**Figure 3B**, black columns).

### Effect of MnTMPYP on $O_{2}^{•–}$ Formation

**Figure 3B** (cross-hatched columns) demonstrates that a SOD mimetic MnTMPYP provided a complete protection in all three models, the intensity of the fluorescent signal did not differ from that in the corresponding control animals. Since we demonstrated in our previous study (Folbergrová et al., 2012) that the difference between group with saline alone and those with saline and MnTMPYP did not differ, we have not studied this group, i.e., saline plus MnTMPYP, in the present study.

### Mitochondrial Respiratory Chain Complex Activities

#### Complex I

**FIGURE 4:**
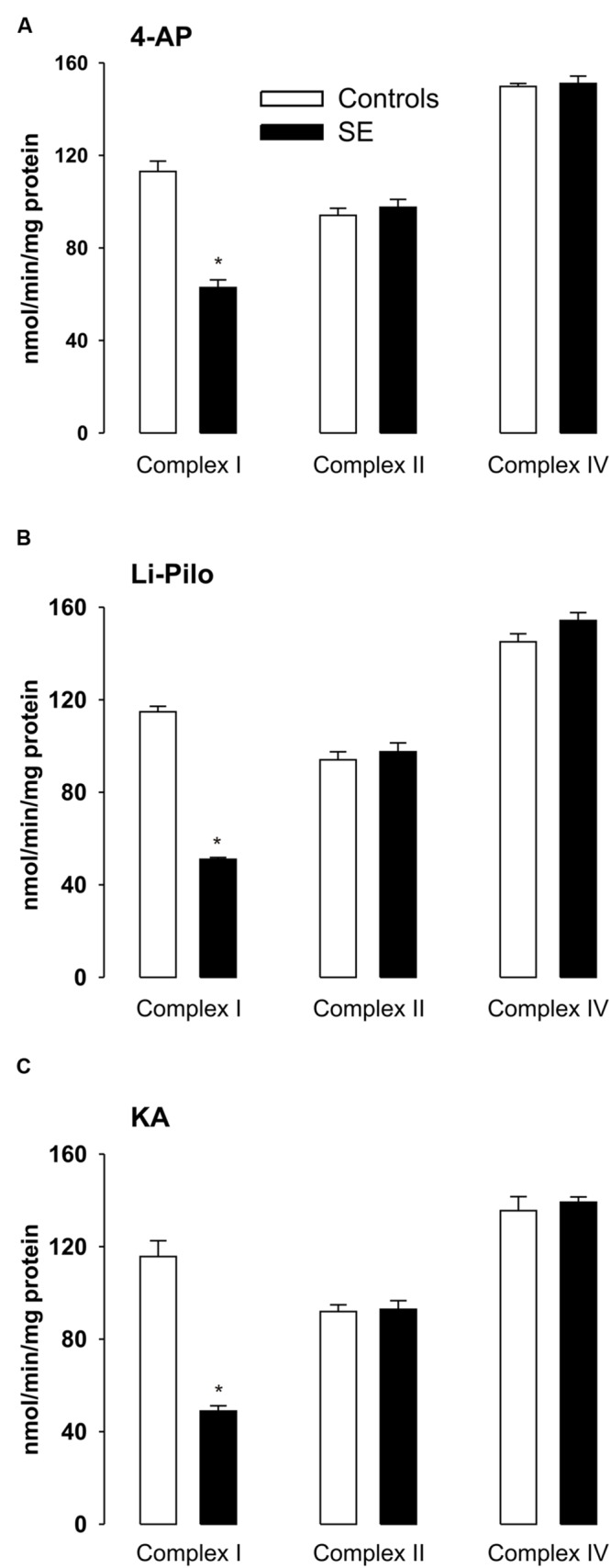
**(A–C)** Activities of complex I, complex II, and complex IV in mitochondria isolated from cerebral cortex of immature rats at ∼ 20 h of survival following SE induced by 4-AP **(A)**, Li-Pilo **(B)**, and KA **(C)** (for more details see Materials and Methods). White columns, control animals; black columns, seizure groups. Results are expressed as nmol/min/mg protein. Mean values for 6–12 mitochondrial preparations ± SEM. ^∗^*P* < 0.05 as compared with control animals.

As shown in **Figures 4A–C**, seizures induced in immature rats by all three convulsant drugs lead to a substantial decrease of complex I activity, corresponding to more than 50%. This decrease could already be detected during the acute phase of seizures (data not shown) and it persisted to the same extent at ∼20 h of survival following the acute phase of seizures. The same decrease of complex I activity was evident when expressed as a ratio to citrate synthase (data not shown).

The observed inhibition was selective for complex I, since the activities of complexes II and IV remained in all three models in the control range (**Figures 4A–C**).

### Complex I Activities at Various Time Intervals of Survival after Acute Phase of Seizures

**FIGURE 5:**
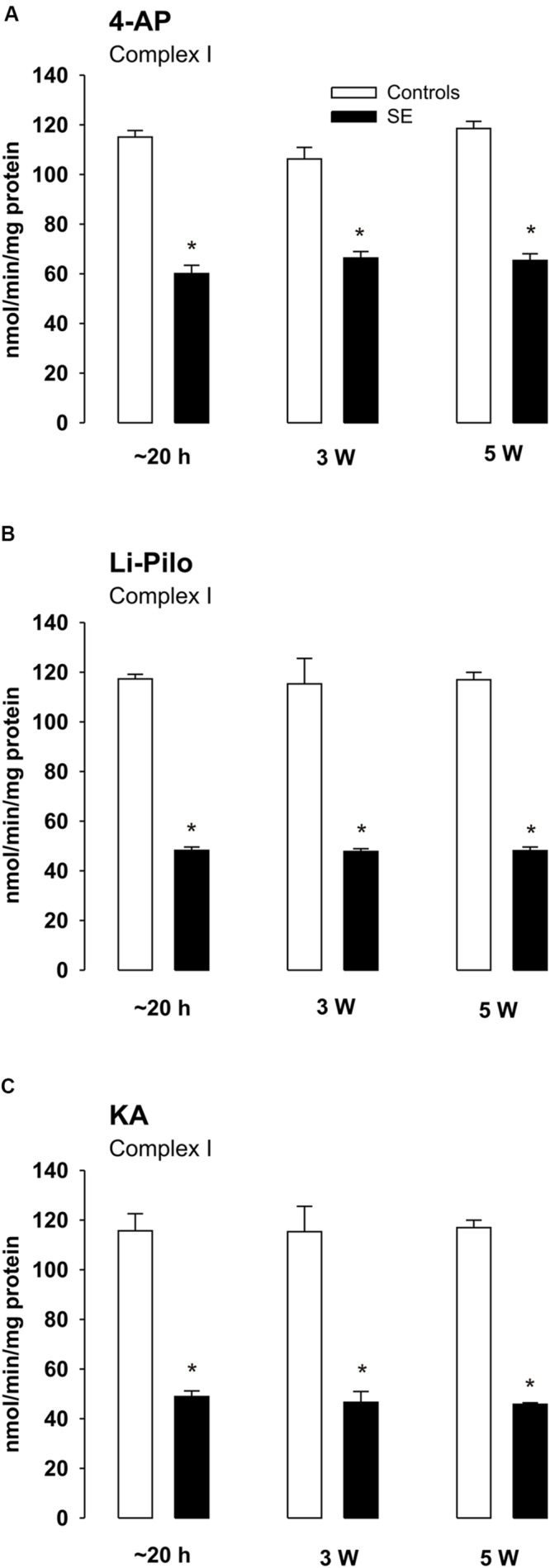
**(A–C)** Complex I activity in mitochondria isolated from the cerebral cortex of immature rats at ∼20 h, 3 and 5 weeks, respectively, of survival following SE induced by 4-AP **(A)**, Li-Pilo **(B)**, and KA **(C)** (for more details see Materials and Methods). White columns, control animals; black columns, seizure groups. Results are expressed as nmol/min/mg protein. Mean values for 6–12 mitochondrial preparations ± SEM. ^∗^*P* < 0.05 as compared with appropriate controls.

The decrease of complex I activity persisted to the same extent also during the long periods of survival studied (up to 5 weeks; **Figures 5A–C**). Thus, no recovery of the inhibited enzyme activity could be detected. Similarly as at a short time of survival, the inhibition was selective for complex I, the activities of complexes II and IV remained during the whole period studied in the control range (data not shown).

### Effect of Free Radical Scavengers on Complex I Inhibition

**FIGURE 6:**
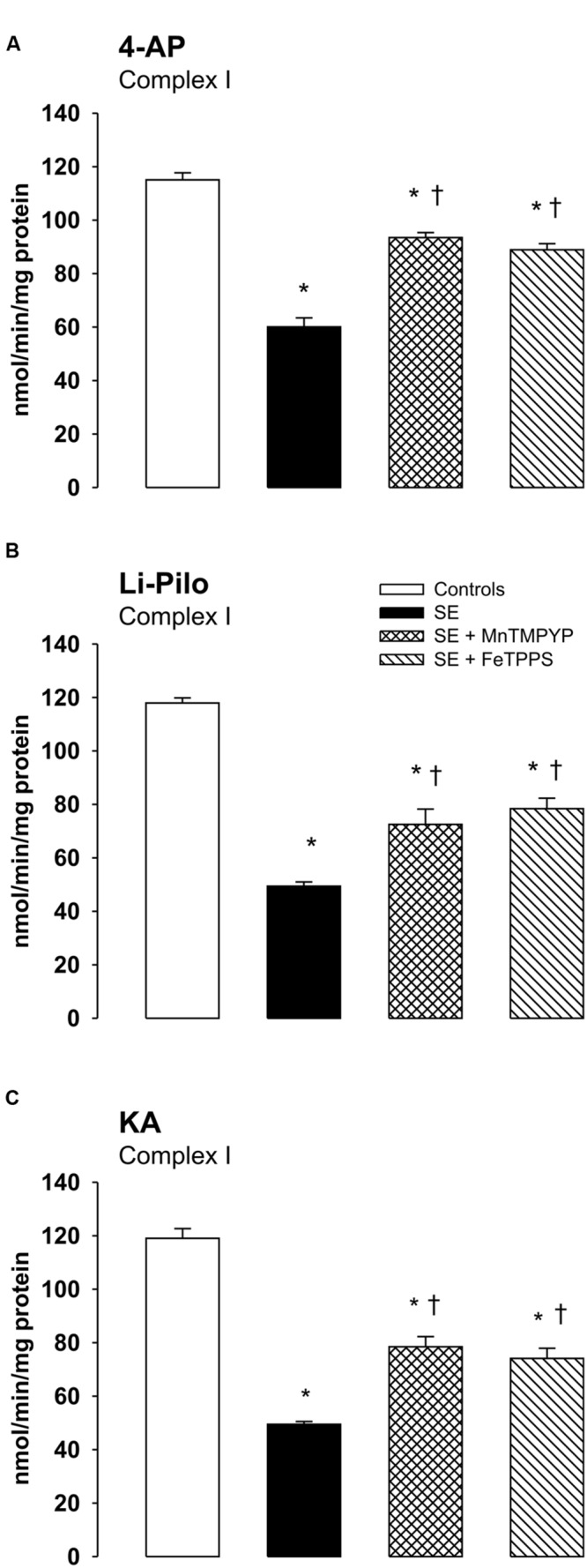
**(A–C)** Effect of MnTMPYP (SOD mimetic) and FeTPPS (a selective peroxynitrite scavenger) on complex I inhibition. Mitochondria were isolated from the cerebral cortex of immature rats at ∼20 h of survival after SE induced by 4-AP **(A)**, Li-Pilo **(B)**, and KA **(C)**. MnTMPYP and FeTPPS were given by i.p. injections in two doses; MnTMPYP (3 mg/kg each) 30 min prior and 30 min after application of convulsant compounds; FeTPPS (10 mg/kg each) 90–120 min prior and 6–10 min after application of convulsant compounds. Results are expressed as nmol/min/mg protein. Mean values for 4–6 mitochondrial preparations ± SEM. ^∗^*P* < 0.05 as compared with the controls; ^†^*P* < 0.05 as compared with convulsants alone.

As evident in **Figures 6A–C**, both SOD mimetic MnTMPYP and a selective peroxynitrite scavenger FeTPPS provide a marked attenuation of complex I inhibition in all three models studied. The protection was significant, but not complete, as complex I activities in groups with MnTMPYP and/or FeTPPS remained significantly lower as compared with control animals.

### Evaluation of Brain Damage

Brain damage, evaluated at 24 h of survival, though rather small was evident in all three models of SE, but the distribution and the extent somewhat differed, as it is demonstrated in **Table 1**. In animals surviving 4-AP SE, the degenerated neurons were found in several thalamic nuclei, particularly in the mediodorsal and in the reuniens nucleus, in the hippocampal fields CA1, CA3 and in the septum. Negative findings concern the DG.

**Table 1:** Degenerated (Fluoro-Jade B positive) neurons, semiquantitatively evaluated in the selected structures: 0-4 neurons per area of evaluated structure = 0; 5-10 neurons = 1; 11-20 neurons = 2; 21-40 neurons = 3; more than 40 = 4; for more details see section Material and Methods.

Structure	4-AP	Li-Pilo	KA
MD	1.67 ± 0.24	2.50 ± 0.72	0.11 ± 0.11
LD	0.78 ± 0.28	0.50 ± 0.34	0.00 ± 0.00
LP	0.44 ± 0.18	0.50 ± 0.50	0.00 ± 0.00
Re	2.00 ± 0.47	0.67 ± 0.42	0.00 ± 0.00
VL	0.56 ± 0.18	0.83 ± 0.48	0.00 ± 0.00
VPL	0.56 ± 0.24	0.00 ± 0.00	0.00 ± 0.00
VM	0.44 ± 0.24	0.17 ± 0.17	0.00 ± 0.00
Po	0.56 ± 0.24	0.00 ± 0.00	0.00 ± 0.00
ANT	0.22 ± 0.15	0.17 ± 0.17	1.11 ± 0.39
CA1 septal	1.11 ± 0.31	2.50 ± 0.50	0.00 ± 0.00
CA3 septal	1.22 ± 0.32	0.33 ± 0.33	0.11 ± 0.11
CA1 temporal	1.44 ± 0.53	2.00 ± 0.68	2.00 ± 0.33
CA3 temporal	0.89 ± 0.42	0.17 ± 0.17	0.78 ± 0.32
DG	0.00 ± 0.00	0.17 ± 0.17	0.00 ± 0.00
Septum	1.33 ± 0.29	0.83 ± 0.31	1.78 ± 0.28
Amygdala	0.00 ± 0.00	1.50 ± 0.50	0.00 ± 0.00
Hypothalamus	0.00 ± 0.00	0.00 ± 0.00	0.00 ± 0.00

In Li-Pilo model, the degenerated neurons were found in several thalamic nuclei, particularly in the mediodorsal nucleus. In the hippocampus, the field CA1 was more affected as compared to both the CA3 and the DG. Moderate neuronal degeneration was observed in the amygdala, localized in the medial and mediobasal nucleus.

In KA model, only minimal neuronal lesion was demonstrated within the thalamus, with degenerated neurons particularly in the anterior group of thalamic nuclei (anteromedial and anteroventral nucleus). In the hippocampus the both subdivisions of fields CA1 and CA3 were affected. Negative finding concerned DG. In the septum degeneration was found only in the lateral septal nuclei.

## Discussion

The major finding of the present study is the evidence of oxidative stress and mitochondrial dysfunction in the brain of immature rats during SE. These findings on SE induced by three substances with a different mechanism of action used in the present study together with our previous results on HCA model (Folbergrová et al., 2010, 2012) prove that oxidative stress in immature brain during SE does occur and can be thus considered a general phenomenon.

All three substances, with the doses employed, induced in immature rats seizures that had character of SE, but both the behavior and EEG pattern differed substantially.

The important findings concern the effect of MnTMPYP and FeTPPS, demonstrating no anticonvulsant effect. The observed effects provided by these scavengers (normalization of $O_{2}^{•–}$ levels and a significant protection of complex I activity) can thus be considered due to their antioxidant properties.

Previous data demonstrated that the immature rat brain has a high ability to compensate for the increased energy demands associated with seizure activity, most likely due to the increased glycolysis (Folbergrová, 1993, 1997; Folbergrová et al., 2000). The present data confirm that SE in all three models studied is also associated with a pronounced increase of glycolysis, but in 4-AP- and Li-Pilo–induced SE, this was apparently not sufficient to compensate completely for the increased energy demand, as evident from a small, but significant decreases of PCr levels (**Figure 2A**). In KA model, ATP and PCr levels did not change in spite of only a small increase of glycolysis. It can be assumed that the observed differences may reflect different intensities of seizures occurring in the individual models.

In the present study Het method has been employed for $O_{2}^{•–}$ detection and, the fluorescent red signal of the oxidized products of Het (reflecting $O_{2}^{•–}$ production) was evaluated microscopically. It should be mentioned that several limitations attributed to the Het assay have been reported recently (Zielonka and Kalyanaraman, 2010; Kalyanaraman et al., 2014). Mainly, Het reacts not only with $O_{2}^{•–}$ to form 2-hydroxyethidium as the major and specific product, but it also reacts with other ROS and/or oxidants to form ethidium. Fluorescent signal reflects most likely both these products since to distinguish them by fluorescence microscopy is almost impossible (Folbergrová et al., 2012). In support for the involvement of $O_{2}^{•–}$ are our data demonstrating the complete prevention of the increased fluorescent signal after the treatment animals with SOD mimetic MnTMPYP (**Figure 3B**). Despite the mentioned limitations the Het method can be considered a good marker of oxidative stress (Zielonka and Kalyanaraman, 2010).

Mitochondrial dysfunction in relation to temporal lobe epilepsy has been demonstrated in humans (Kunz et al., 2000; Kann et al., 2005; Lee et al., 2008) and in several experimental models of epilepsy in adult animals (Waldbaum and Patel, 2010; Folbergrová and Kunz, 2012). Our previous (Folbergrová et al., 2007, 2010) and the present results clearly indicate that similar signs of mitochondrial dysfunction also occur in immature brain following SE.

In all three models studied, a marked decrease (∼50%) of complex I activity that was selective for complex I was observed. In HCA model, by performing electrophoretic (BN-PAGE and SDS-PAGE) and Western blot (WB) analysis, using antibodies against several subunits of complex I, we have shown that the decrease was not associated with changes in the size of the assembled complex I or with changes in mitochondrial content of complex I. Concurrently with the decreased activities of complex I, significant increases in three markers of mitochondrial oxidative damage (3-NT, 4-HNE and protein carbonyls) were detected (Folbergrová et al., 2010). On the basis of all these findings it was proposed that oxidative modification (inactivation) of complex I, localized potentially on some critical subunit, is very likely responsible for the deficiency of complex I activity, in accordance with extreme sensitivity of this enzyme to both oxidative and nitrosative stress (Folbergrová et al., 2010 and references therein). Due to the increased ROS production detected in all three models, it seems likely that there are conditions favoring oxidative modification of sensitive targets. Several posttranslational oxidative modifications of complex I can occur, such as carboxylations, nitration of tyrosine (and/or tryptophane) residues, *S*-nitrosation of some of its protein thiols etc. (Folbergrová et al., 2010; Ryan et al., 2012, 2014; Folbergrová, 2013). Potential role of other factors beside oxidative inactivation can not, however, be excluded. The involvement of oxidative modification of complex I is supported by our findings demonstrating that the decrease of complex I activity could be significantly attenuated by SOD mimetics MnTMPYP (**Figures 6A–C**) or Tempol (Folbergrová et al., 2011) and also by a selective peroxynitrite scavenger and decomposition catalyst FeTPPS (**Figures 6A–C**).

Importantly, in agreement with our previous study concerning HCA-induced SE (Folbergrová et al., 2010), the profound decrease of mitochondrial complex I activity persists in all three models studied during the long periods of survival, up to 5 weeks (the longest period studied), i.e., periods during which development of spontaneous seizures (epileptogenesis) may occur.

There is a question what may be potential consequences of persisting inhibition of complex I activity. It should be emphasized that complex I is not only a target for ROS and RNS, but it is also the important source of their production, especially when partially inhibited (Sipos et al., 2003; Kudin et al., 2004; Kussmaul and Hirst, 2006; Fato et al., 2008; Parihar et al., 2008). It has been reported that overproduction of ROS caused by complex I inhibition may be responsible for triggering the activation of specific kinase pathways, for the release of cytochrome c and other proapoptotic factors and finally for cell death (Perier et al., 2005; Marella et al., 2007; Ott et al., 2007).

Another possibility to be considered is a potential contribution to epileptogenesis. It can be speculated that an increase of oxidative stress as a consequence of complex I inhibition could lead to the increased excitability. It has been shown that high-affinity astroglial and neuronal glutamate transporters, which are important for the maintaining low levels of synaptic glutamate, are extremely sensitive to oxidative damage (Trotti et al., 1998). It should be mentioned in this connection that mice partially deficient in SOD2 (SOD2^-/+^; a model of sublethal chronic elevation of mitochondrial $O_{2}^{•–}$) have increased seizure susceptibility (Liang and Patel, 2004).

Altogether, inhibition of complex I can lead to the enhanced production of ROS and/or RNS which may contribute not only to neuronal injury, but also to epileptogenesis.

Mild brain damage, evaluated at 24 h of survival following the acute phase of SE, was detected in all three models studied, even though the distribution and the extent was somewhat different (see **Table 1**). SE induced by 4-AP leads to larger changes than KA- or Li-Pilo-induced SE. It is worthwhile notion that employing Fluoro-Jade B method, small injury was detected also in KA model in contrary to previous studies claiming that KA SE in immature rats of less than 18 days of age does not lead to injury (Nitecka et al., 1984). It should be emphasized that brain damage in all three models was rather small as compared with massive neuronal degeneration following SE induced by HCA (Folbergrová et al., 2005). In the latter case brain injury most likely reflects not only intense seizure activity, but also the neurotoxic effect of HCA. Importantly, in spite of the existing differences in brain injury, the extent of oxidative stress as discussed above is more or less comparable in all the four mentioned models of SE (Folbergrová et al., 2012 and the present results). This suggests that the extent of brain injury is apparently not a decisive factor for the extent of oxidative stress.

Thus, in spite of the reported differences production of ROS and oxidative stress was evident and comparable in all three models studied. This suggests that the existence of oxidative stress does not depend on the substances used for induction of SE and it is a common consequence of SE.

## Conclusion

This study reveals that excessive oxidative stress induced by SE is much more general phenomenon than previously considered. The oxidative stress-related functional, morphological, and biochemical changes following the SE in adult brain are also present during brain development. This finding may open new prospects for the development of more precise treatments, by targeting the mechanisms, which play a causal or additive role in the pathogenesis of epilepsies occurring in infants and children.

## Author Contributions

JF: Experimental design, biochemical measurements, writting of the manuscript. PJ: Analysis of mitochondrial metabolism. HK: Performed SE models and behavioral assesment. RD: Morphological evaluations. JO: Experimental design, electrophysiology, fluorescence measurements, and co-writting of the manuscript.

## Conflict of Interest Statement

The authors declare that the research was conducted in the absence of any commercial or financial relationships that could be construed as a potential conflict of interest.

## Abbreviations

3-NT: 3-nitrotyrosine
4-AP: 4-aminopyridine
4-HNE: 4-hydroxynonenal
ANT: anterior thalamic nuclear group
DG: Dentate gyrus
FeTPPS: 5,10,15,20-tetrakis (4-sulfonatophenyl) porphyrinate Iron (III)
HCA: homocysteic acid
Het: hydroethidine
KA: kainic acid
LD: laterodorsal thalamic nucleus
Li-Pilo: lithium-pilocarpine
LP: lateral posterior thalamic nucleus
MD: mediodorsal thalamic nucleus
MnTMPYP: Mn (III) tetrakis (1-methyl-4-pyridyl)porphyrin pentachloride
PCr: phosphocreatine
Po: posterior thalamic nuclear group
Re: reuniens thalamic nucleus
RNS: reactive nitrogen species
ROS: reactive oxygen species
$O_{2}^{•–}$: superoxide anion
SE: status epilepticus
SOD: superoxide dismutase
VL: ventrolateral thalamic nucleus
VM: ventromedial thalamic nucleus
VPL: ventral posterolateral thalamic nucleus
